# Hair Cortisol Is Associated With Social Support and Symptoms in Schizophrenia

**DOI:** 10.3389/fpsyt.2020.572656

**Published:** 2020-09-24

**Authors:** Fuzhong Yang, Xinyi Cao, Xiujia Sun, Hui Wen, Jianyin Qiu, Hua Xiao

**Affiliations:** ^1^ Shanghai Mental Health Center, Shanghai Jiaotong University School of Medicine, Shanghai, China; ^2^ State Key Laboratory of Microbial Metabolism, Joint International Research Laboratory of Metabolic & Developmental Sciences, School of Life Sciences and Biotechnology, Shanghai Jiao Tong University, Shanghai, China

**Keywords:** schizophrenia, hair cortisol concentrations, childhood trauma, stressful life events, social support

## Abstract

**Introduction:**

Psychosocial stressors may worsen psychotic symptoms in schizophrenia, while social support could protect against the effects of stress in schizophrenia. Hypothalamus-pituitary-adrenal axis dysfunction has been associated with schizophrenia. Hair cortisol concentrations (HCC) allow assessment of cumulative cortisol secretion over the preceding 3 months. The relationship between HCC, psychosocial stressors, social support, and the clinical characteristics of schizophrenia needs to be explored.

**Methods:**

One hundred nine schizophrenia patients and 86 healthy controls between the ages of 18 and 60 were enrolled in the study. Three-centimeter samples of hair were collected from the scalp and HCC were measured using ELISA kits. Linear regression and factor analysis were employed to examine the relationship between HCC, childhood trauma, the number of stressful life events (SLE), the amount of social support in the 3 months prior to the hair cortisol assessment and clinical characteristics of schizophrenia.

**Results:**

Schizophrenia patients experience more SLE in their lifetime, receive less social support, and have lower HCC in the recent 3 months compared to healthy controls. In the schizophrenia patients, HCC are positively associated with the amount of social support and negatively associated with the severity of delusions. The interaction between social support and SLE predicts decreased HCC. Factor analysis shows that a subgroup of schizophrenia patients who experience childhood trauma and SLE are characterized by decreased HCC.

**Conclusions:**

Findings indicate social support could be a moderator for the relationship between SLE and HCC which may attenuate the effects of SLE in schizophrenia.

## Introduction

Psychosocial stressors, such as childhood trauma or stressful life events (SLE), appear to play a significant role for the onset and course of schizophrenia. They have been found to prospectively predict psychotic symptoms exacerbation and be associated with increased risk of relapse (1–3). These data support the neural diathesis-stress model of schizophrenia which implicate a role for stress in the etiology of schizophrenia (4, 5). Social support is usually defined as the existence or availability of people on whom we can rely, people who let us know that they care about, value, and love us (6). Social support can help individuals to cope with everyday life, particularly in response to critical situations. Increased subjective social support shows correlation with a lower degree of psychotic symptoms (7, 8). Social support can provide a buffer against psychosocial stressors and protect against the negative effects of SLE in schizophrenia (7, 9). Furthermore, the beneficial effects of social support on health can be influenced by culture and race (10, 11). The relationship between psychosocial stressors and social support in schizophrenia has seldom been investigated in the Han Chinese population.

Cortisol are essential for an adequate response to stress. Cortisol concentrations have been recognized as the inner indicator of stress response regulated by the hypothalamic-pituitary-adrenal (HPA) axis (12). Abnormal HPA axis functioning in the form of altered cortisol concentrations has been associated with more severe symptoms in schizophrenia (13). Studies have either shown no difference, elevated or attenuated cortisol concentrations in schizophrenia compared to healthy controls (13–16). Meanwhile, both hypo- and hyper-function of cortisol response to stress have been reported in schizophrenia (16–18). An impaired function of the glucocorticoid receptor-mediated negative feedback may account for the dysregulated HPA axis [8]. The inconsistent findings about the cortisol concentrations in schizophrenia could partly be caused by methodological differences between studies, but could also be caused by differential exposure to the number of SLE and the amount of received social support of the participants enrolled in studies. Studies report psychosocial stressors and social support are inversely associated with cortisol concentrations. For example, elevated cortisol concentrations are not only associated with psychosocial stressors (19), but also with lower positive social support (20). Few studies examine the relationship between social support, psychosocial stressors, and cortisol concentrations in schizophrenia.

Cortisol measurements in saliva and plasma are validated measures of acute stress, but they are subject to the normal diurnal variation in cortisol secretion, and easily affected by temporary or transient disturbances in psychosocial stress on the day of measurement (21, 22). In contrast, hair cortisol concentrations (HCC) can reflect long-term cumulative cortisol secretion and chronic stress response for periods of several months. Typically, the average hair grows 1 cm per month, so a 3 cm segment of scalp hair is assessed to determine cortisol secretion during the preceding 3 months (23). As a measure of cortisol changes over time, HCC are suitable for the evaluation of cumulative effects of stress. Thus, HCC may be robust biomarkers in trauma and significant stress-related mental disorders, such as schizophrenia (24). Yet, studies investigating HCC in schizophrenia are scarce and the results are inconclusive (25, 26).

The purpose of this study is to investigate the associations between childhood trauma, the number of SLE, the amount of social support in the 3 months prior to the hair cortisol assessment, clinical characteristics and HCC in schizophrenia patients compared to healthy controls. We also explored the factorial constructs of HCC, psychosocial stressors, social support and symptoms in schizophrenia patients. We hypothesize that schizophrenia patients experiencing more psychosocial stressors and receiving less social support may exhibit more severe symptoms and HPA axis dysfunction indexed by abnormal HCC.

## Materials and Methods

### Sample

The patients were recruited consecutively from Shanghai Mental Health Center inpatient department. A total of 138 schizophrenia patients were assessed for eligibility and 21 of them did not meet the inclusion criteria and were excluded. Among the 117 eligible schizophrenia patients, 8 of them refused to participate. A total of 109 patients and 86 healthy control subjects were enrolled in the study. Psychiatrists and psychiatrists-in-training performed clinical assessments. All clinical personnel completed training in diagnostics and symptoms rating. Inclusion criteria for patients included age range between 18 and 60 years, had a diagnosis of schizophrenia according to Diagnostic and Statistical Manual of Mental Disorders (DSM-IV-TR) (27). They were required to have a total Positive and Negative Syndrome Scale (PANSS) score between 60 and 120.

Controls with similar ethnic background were recruited by means of advertisements in and around the hospital in the same district in Shanghai. Inclusion criteria for the healthy controls were the following: being between 18 and 60 years and having no lifetime diagnosis of psychiatric disorder. Exclusion criteria for all groups included the following: neuroendocrine disorders, neurological disorders, any use of medication that may influence HPA axis function, organic psychosis, unstable or uncontrolled medical conditions interfering with brain function. Moderate to severe brain damage or IQ under 70 were also exclusion criteria for all participants.

All participants were interviewed using a pencil-and-paper version of the interview on average one hour for the patients and 30 minutes for the controls. All participants were informed of the purpose of the study and a written informed consent was established before the interview. The participants were enrolled between January 2018 and December 2019. The ethics committee in Shanghai Mental Health Center approved this study (registration number 2018-13).

### Measures

Diagnoses were based on the Structured Clinical Interview for DSM-IV-TR axis I disorders (SCID-I) (28). Psychotic symptoms were rated using PANSS. Items are divided into three symptoms domains which include positive symptoms, negative symptoms and general psychopathology (29). Formal thought disorder was assessed using the Thought, Language, and Communication scale (TLC) (30). Briefly, the TLC contains 18 items and an overall rating (global TLC). Severity ratings of the items 1–9 range from 0 (absent) to 4 (extreme), while severity ratings of the items 10–18 range from 0 (absent) to 3 (severe). Symptom severity and function during the previous three months were rated using the Global Assessment of Functioning Scale (GAF) (31). The GAF evaluates both symptom severity and functioning, ranking a patient from 1 (lowest score) to 100 (highest score) on both scales.

The instrument for assessment of SLE was translated from the interview employed in the Virginia Adult Twin Study of Psychiatric and Substance Use Disorders (VATSPUD) study, which assessed thirteen negative SLE (32). The thirteen stressful personal events include loss of confidant (death of a spouse, child or sibling), marital difficulties (divorce or marital separation), job loss (laid off from a job or being fired), major financial crisis, legal problems (problems with police or other legal trouble), serious illness, life-threatening accident, natural disaster (fire, flood, etc.), witness of someone being injured or killed, assault (physical assault, rape), and threat (captive, kidnap). The timing of SLE is recorded if the coding is positive.

We assessed childhood emotional neglect (CEN), childhood physical abuse (CPA), and childhood sexual abuse (CSA) through the questions adapted from the Childhood Trauma Questionnaire (CTQ) (33). “Before the age of 16 years old, were you ever sexually abused as a child?”, “Before the age of 16 years old, were you ever physically abused as a child?”, “Before the age of 16 years old, were you ever seriously neglected as a child?”. Sexual abuse refers to any unwanted incidents such as (1) inviting or requesting the child to do something sexual, (2) touching or fondling private parts, (3) making them touch the person in a sexual way, or (4) attempting or having sexual intercourse. Physical abuse refers to bodily assaults on a child by an older person that pose a risk of, or result in, injury. Emotional neglect refers to a lack of emotional support and inadequate attention to a child’s emotional needs, including the need for affection.

Social support was measured using the 6-item short form of the Social Support Questionnaire (SSQ) (34), a psychometrically sound and conveniently administered instrument. The items have two parts. The first part of each item assesses the number of available others the participant has, whom he/she can rely on in times of need. In the second part of the items, the participant is asked to indicate on a 6-point Likert scale how satisfied he/she is with the overall support from the number of people indicated in the first part ranging from 1 (Very dissatisfied) to 6 (Very satisfied).

The age at onset of schizophrenia was assessed retrospectively by reviewing the medical record and was defined as the age at which the first manifestation of psychotic symptoms fulfilling the criteria of schizophrenia occurred. Symptoms reported during the recent episode were regarded as positive and classified according to DSM-IV-TR diagnostic criteria. The duration of illness was defined as the time between the age at onset and age at interview of schizophrenia. The risk of relapse was defined as the ratio between numbers of episodes in lifetime and duration of illness. Medication compliance was defined as the proportion of total duration of medicine-taking over the total duration of illness.

### Hair Sample and Hair Cortisol Analysis

Hair sample preparation has been described in detail elsewhere (35). A 3 cm hair segment, approximately 100–150 hairs were cut from the posterior vertex as close to the scalp as possible from the participants. The hairs were cut and stored in envelopes until preparation. As hair grows at an average speed of 1 cm per month, this 3 cm segment can be assumed to reflect cortisol secretion during the preceding 3 months (36). The 3 cm hair segments were minced with small surgical scissors and 50 mg of powered hair were weighed and separated into a glass vial. One ml of methanol was added to extract cortisol from the hair samples and incubated for 16 h at 52°C while gently shaking. Afterwards, the methanol was transferred to a clean glass vial and was evaporated under a constant nitrogen stream until completely dry. The samples were then dissolved in 250 μl phosphate buffered saline (pH 8.0) and vortexed until thoroughly mixed. Cortisol concentrations in the hair extracts were measured using a commercial ELISA kit for salivary cortisol (DRG Instruments GmbH, Marburg, Germany) according to the manufacturer’s instructions. HCC values are presented in pg/mg hair.

### Statistics

The statistical analyses were completed using R (version 3.3.1) (37). The descriptive statistics are presented as percentages for discrete variables and as means (standard deviation, S.D.) for continuous variables. T- test or Chi - square test were used to compare HCC and social demographic features between schizophrenia and healthy controls. We applied linear regression to examine the association of HCC, treated as a continuous independent variable, with clinical characteristics of schizophrenia and different phenotypes. Coefficient values were used to quantify the strength of associations. The statistical significance for all tests was set at *P* < 0.05 as the analyses were exploratory in nature.

To examine the relationship and factorial constructs of HCC, psychosocial stressors, social support and symptoms in schizophrenia patients, exploratory factor analysis was performed by using both varimax and promax rotations. Interpretations of the scree plot and eigenvalue were used to guide decisions on the number of factors to be extracted. Factor loadings ≧ 0.30 were considered to be substantial.

## Results

The average age of schizophrenia at interview was 40.8 (S.D. = 12.2) years (range 18–60). The mean age at onset of schizophrenia was 27.7 (S.D. = 11.4) years (range 18–55). The average age of healthy controls at interview was 42.2 (S.D. = 10.9) years (range 18–60). Schizophrenia patients have significantly lower HCC (14.2 pg/mg, S.D. = 11.7) than healthy controls (18.5 pg/mg, S.D. = 10.5) (*P* < 0.05).

**Table 1** shows the results for socio-demographic features of schizophrenia patients and healthy controls. Schizophrenia patients were less likely to be employed, to be married and to receive education (all *P* < 0.001).

**Table 1:** Socio-demographic variables between schizophrenia and healthy controls.

	Schizophrenia (Number = 109, %)	Healthy controls (Number = 86, %)	Statistics	*P*-value
Gender				
Male	51(46.8)	38(44.0)	*X* ^2^ = 0.04	0.83
Female	58(53.2)	48(56.0)		
Marital status				
Married	45(41.3)	74(86.0)	*X* ^2^ = 42.1	**<0.001**
Separated/divorced	12(11.0)	0		
Widowed	1(0.9)	0		
Never married	51(46.8)	12(14.0)		
Education				
Primary school	5 (4.6)	6 (7.0)	*X* ^2^ = 34.6	**<0.001**
High school	39(35.8)	30(35.0)		
Technical school	40 (36.7)	6(7.0)		
Bachelor	24(22.0)	32(37.0)		
Master or PhD	1 (0.9)	12(14.0)		
Employment				
Working for pay	16 (14.7)	74(86.0)	*X* ^2^ = 115.5	**<0.001**
Retired	28(25.7)	2(2.0)		
Laid off and looking for work	56(51.4)	0(0)		
Other	7(6.4)	6(7.0)		
Keeping house	2(1.8)	4(5.0)		

The bold values are significant with a p-value <0.05.

The differences of SLE, childhood trauma, social support and body mass index between schizophrenia patients and healthy controls are presented in **Table 2**. Compared with healthy controls, schizophrenia patients had more SLE in lifetime, less people who totally accepted them, less people whom they could count on to be consoled and less average number of available others (all *P* < 0.05). There was a trend toward significance that schizophrenia patients experienced more CEN compared to healthy controls (*P* = 0.08).

**Table 2:** Comparison and association between variables and HCC.

Variables	Variables		Association of HCC with variables			
	Schizophrenia patients(Number = 109)	Healthy controls(Number = 86)	Schizophrenia patients (Number =109)		Healthy controls(Number = 86)	
		
	Mean (SD)	Mean (SD)	Coefficient ± SE	*P*	Coefficient ± SE	*P*
SLE in lifetime	1.37 (2.83)*	0.6 (0.88)	-1.88 ± 1.08	0.08	0.88 ± 1.87	0.64
SLE in recent 3 months	0.04 (0.19)	0.02 (0.15)	-3.01 ± 5.99	0.62	0.00 ± 10.72	1.00
CEN	0.16 (0.77)	0.02 (0.15)	-0.37 ± 2.07	0.86	-5.73 ± 10.68	0.60
CPA	0.09 (0.59)	0.14 (0.35)	-3.46 ± 5.90	0.56	-1.45 ± 4.67	0.76
CSA	0.06 (0.50)	0	-3.73 ± 8.39	0.66	NA	NA
Number of available others in average	1.87 (1.23)*	2.38 (1.21)	3.38 ± 1.40	**0.02**	-0.86 ± 1.36	0.53
Satisfaction with social support	32.43 (2.87)	31.81 (3.42)	0.02 ± 0.48	0.96	-0.12 ± 0.49	0.81
Number of people count on to be dependable	2.43 (1.48)	2.91 (1.84)	2.28 ± 0.87	**0.01**	-0.74 ± 0.89	0.41
Number of people count on to help you feel more relaxed	1.76 (1.54)	2.1 (1.32)	1.75 ± 0.96	0.07	-0.80 ± 1.25	0.52
Number of people accepts you totally	1.75 (1.22)*	2.23 (1.19)	1.01 ± 1.14	0.38	-1.97 ± 1.34	0.15
Number of people count on to care about you	2.01 (1.24)	2.14 (1.13)	2.62 ± 1.03	**0.01**	0.41 ± 1.45	0.78
Number of people count on to help you feel better	1.84 (1.35)	2.23 (1.38)	1.10 ± 1.03	0.29	-0.41 ± 1.19	0.73
Number of people count on to console you	1.71 (1.28)*	2.53 (2.27)	1.65 ± 1.12	0.15	0.15 ± 0.72	0.83
BMI	23.29 (3.67)	23.18 (2.55)	-0.10 ± 0.34	0.77	0.47 ± 0.64	0.47

HCC, hair cortisol concentrations; SLE, stressful life events; CEN, childhood emotional neglect; BMI, body mass index; CSA, childhood sexual abuse; CPA, childhood physical abuse; SD, standard deviation; SE, standard error, *P < 0.05 (compared with healthy controls). The bold values are significant with a p-value <0.05.

We examined the association between psychosocial stressors, social support, body mass index, and HCC both in schizophrenia patients and healthy controls (**Table 2**). In the schizophrenia patients, HCC were positively associated with the average number of available others (*P* = 0.02), including the number of people they can count on to be dependable (*P* = 0.01) and count on to be cared about (*P* = 0.01). There was a trend that decreased HCC were associated with more SLE (*P* = 0.08). HCC were not associated with SLE that happened within the past 3 months (*P* = 0.62). While in the control group, HCC were not associated with psychosocial stressors, social support and BMI (all *P* > 0.05).

The amount of social support (average number of available others) was calculated as a moderator for the relationship between psychosocial stressors and HCC (**Table 3**). In the schizophrenia patients, social support was positively associated with HCC in all of the moderation models. The amount of social support predicted HCC (all *P* < 0.05). SLE and childhood trauma did not predict HCC (all *P* > 0.05). The interaction term between social support and SLE was significant and predicted decreased HCC [F _(3,89)_ = 3.64, *P* = 0.02]. The interaction terms between social support and CEN [F _(3,89)_ = 2.00, *P* = 0.12], between social support and CPA [F _(3,89)_ = 2.87, *P* = 0.06], between social support and CSA [F _(3,89)_ = 1.80, *P* = 0.15] were not significant. In the control group, the interaction terms between social support and SLE, between social support and childhood trauma were not significant (all *P* > 0.05).

**Table 3:** Moderation models for HCC.

Moderation models	Schizophrenia (Number = 109)		Healthy controls (Number = 86)	
	Coefficient ± SE	*P*	Coefficient ± SE	*P*
Model 1: social support - SLE				
Social support	6.20 ± 2.08	**0.00**	-2.03 ± 1.15	0.18
SLE	3.37 ± 3.38	0.32	-1.64 ± 3.41	0.63
Social support * SLE	-4.21 ± 1.93	**0.03**	1.02 ± 0.88	0.25
Model 2: social support - CEN				
Social support	2.96 ± 1.27	**0.02**	-0.90 ± 0.95	0.35
CEN	7.96 ± 8.76	0.37	-6.35 ± 7.47	0.40
Social support * CEN	-3.34 ± 3.30	0.32	NA	NA
Model 3: social support - CPA				
Social support	2.94 ± 1.27	**0.02**	-1.03 ± 1.01	0.32
CPA	-5.27 ± 5.81	0.37	-2.81 ± 14.84	0.85
Social support * CPA	NA	NA	1.30 ± 4.47	0.77
Model 4: social support - CSA				
Social support	2.86 ± 1.27	**0.03**	-0.86 ± 0.95	0.37
CSA	13.25 ± 31.24	0.67	NA	NA
Social support * CSA	-8.40 ± 14.64	0.57	NA	NA

HCC, hair cortisol concentrations; SLE, stressful life events; CEN, childhood emotional neglect; CPA, childhood physical abuse; CSA, childhood sexual abuse; SE, standard error. The bold values are significant with a p-value <0.05. The asterisk symbol (*) denotes an interaction between two variables.

We applied linear regression to explore the association between HCC and a series of clinical characteristics in schizophrenia, including symptoms, thought disorder, duration of illness, risk of relapse and social functioning (**Table 4**). HCC were negatively associated with the severity of delusions (*P* = 0.03). We found a trend towards significance for the severity of tension (*P* = 0.06) and uncooperativeness (*P* = 0.08). No other significant associations were found between HCC and all the above mentioned clinical characteristics of schizophrenia.

**Table 4:** Association of HCC with clinical symptoms and characteristics in schizophrenia.

	Mean (SD)	Coefficient ± SE	*P*-value
Clinical symptoms			
Positive scale	22 (4.55)	-0.21 ± 0.26	0.43
Negative scale	16.31 (4.97)	-0.05 ± 0.24	0.84
General pathology	36.98 (6.47)	0.25 ± 0.18	0.17
Total score	75.29 (10.12)	0.05 ± 0.12	0.68
Delusions	4.92 (1.13)	-2.27 ± 1.00	**0.03**
Tension	2.07 (1.29)	1.74 ± 0.93	**0.06**
Uncooperativeness	2.32 (1.57)	1.38 ± 0.78	**0.08**
Thought disorder			
Total score	10.06 (9.11)	0.09 ± 0.15	0.57
Overall rating	1.43 (0.98)	0.99 ± 1.29	0.45
Clinical characteristic			
Duration of illness, years	13.15 (12.51)	-0.04 ± 0.10	0.71
Relapse risk (numbers of relapses/year)	0.69 (0.65)	2.09 ± 2.02	0.30
Medication compliance (%)	59.37 (33.49)	0.02 ± 0.05	0.71
GAF score	76.66 (11.22)	0.15 ± 0.13	0.25

HCC, hair cortisol concentrations; GAF, global assessment of functioning; SD, standard deviation; SE, standard error.

Examination of the scree plot and eigenvalue indicated that a three or four factor solution best fitted the data. We examined both an orthogonal and oblique factor rotation which produced similar results. The orthogonal rotations are more likely to approximate clinical reality (**Table 5**). Factor analysis of the 11 items yielded three factors accounting for 40% of the variance. SLE, CEN, CSA, and HCC loaded prominently on factor I. Positive symptoms, general symptoms and thought disorder loaded substantially on factor II. General symptoms, negative symptoms, satisfaction with social support and CSA loaded the highest on factor III. Items within a factor are supposed to be inter-related. The factor analysis points to a subgroup of schizophrenia patients who experience childhood trauma and SLE are characterized by decreased HCC.

**Table 5:** Exploratory factor analysis of HCC, childhood trauma, social support, and symptoms.

	Factor Ⅰ	Factor Ⅱ	Factor Ⅲ
HCC	-0.31		
Stressful life events	0.97		
Childhood emotional neglect	0.50		
Childhood sexual abuse	0.31		0.36
Childhood physical abuse			
Number of available others in average			
Satisfaction with social support			-0.43
Positive symptoms		0.99	
Negative symptoms			0.51
General symptoms		0.39	0.79
Thought disorder		0.45	

Data indicates factor loadings for orthogonal rotated solution, HCC, hair cortisol concentrations.

## Discussion

Our study aims to examine the relationship between childhood trauma, the number of SLE, the amount of social support, clinical characteristics, and HCC in schizophrenia. We find that schizophrenia patients have lower HCC, experience more SLE, and receive less social support compared to healthy controls. In schizophrenia patients, decreased HCC are significantly associated with less social support and more severe delusions. Social support is observed to be a moderator for the relationship between SLE and HCC in schizophrenia patients. The interaction between social support and SLE predicts decreased HCC. Factor analysis shows that a subgroup of schizophrenia patients who experience psychosocial stressors are characterized by decreased HCC.

Consistent with previous studies, patients with schizophrenia are more likely to have experienced intense psychosocial adversities than healthy controls (26, 38). It has also been shown that schizophrenia patients receive less social support compared to healthy controls (39, 40). Studies report increased subjective social support shows correlation with a lower degree of psychotic symptoms (7), and emotional and socialization supports are helpful for schizophrenia patients (9). On the contrary, exposure to social stress is strongly associated with onset of psychosis, schizophrenia in particular (41). We think it is important to offer social support to schizophrenia patients in rehabilitation programs to improve their quality of life and decrease chances for relapse in their lifetime (9, 42).

The previous studies report mixed results on the cortisol concentrations in schizophrenia patients, including elevated or normal serum or salivary cortisol concentrations compared to healthy controls (13, 15, 16, 43). HCC reflect long-term cumulative cortisol secretion over weeks to months and studies investigating HCC in schizophrenia are scarce. We find schizophrenia patients experience more SLE in their lifetime, yet they have lower HCC than healthy controls. SLE have a negative trend association with HCC in schizophrenia patients. However, one study reports schizophrenia patients with a history of childhood maltreatment have higher HCC relative to healthy controls (25). Another shows no difference in HCC between schizophrenia patients and healthy controls (26). Since social support has been proved to be associated with increased cortisol concentrations (20), the differences in the amount of social support received by the participants may contribute to the inconsistent results in different studies, including ours.

We find more social support is significantly associated with increased HCC in schizophrenia. Furthermore, the interaction between social support and SLE significantly predicts decreased HCC. Thus, social support is observed to be a moderator for the relationship between SLE and HCC in schizophrenia patients. Our results are consistent with previous findings which indicate SLE and social support may influence cortisol concentrations inversely in schizophrenia patients (20). Social support may have protective effects against psychosocial stressors as indexed by HCC, which corroborate the neural diathesis-stress model of schizophrenia (5, 17). The protective effects of social support against psychosocial stressors and its moderating role for the relationship between SLE and HCC could be further investigated by cohort studies.

Besides the cortisol concentrations, cortisol response is another important index reflecting the HPA axis function. Most of the studies report that cortisol stress reactivity is blunted in schizophrenia, showing reduced HPA axis reactivity to stressors, indicating an impaired activation of HPA axis in facing stressors among schizophrenia patients (44, 45). HCC measure long-term cumulative cortisol level and can be interpreted as promising biomarkers of long-term HPA activation (46). Our results show schizophrenia patients experience more psychosocial stressors in their lifetime history, yet they display lower HCC compared to healthy controls. The impaired activation of HPA axis may indicate schizophrenia does not display physiological readiness following psychosocial stressors (47). On the contrary, schizophrenia patients who do not exhibit attenuated cortisol responses to stress may have generally better social functioning (43).

Abnormal HPA axis function and cortisol concentrations have been shown to be associated with the severity of clinical symptoms or the general severity of schizophrenia (16). For example, cortisol concentrations are positively or negatively associated with the severity of negative symptoms, positive symptoms or the severity of a wide array of symptoms in schizophrenia (13, 48, 49). Our results are consistent with the previous findings and show HCC are negatively associated with the severity of delusions and there is a trend association between HCC and tension and uncooperativeness. The severity and exacerbation of symptoms in schizophrenia are associated with cortisol concentrations, yet the underlying mechanism is still unclear. Glucocorticoids receptors are present throughout the central nervous system and thus can mediate the effects of cortisol on several neural systems (50). The synergistic relation between HPA activity and DA neurotransmission can help us to understand how stress exposure leading to increased cortisol secretion might trigger a neuropathological dopamine-driven process (51).

Factor analysis in our study reveals three latent factors within the schizophrenia patients. One subgroup of patients who experience childhood trauma and SLE are characterized by decreased HCC. The second subgroup of patients who experience CSA, exhibit more severe symptoms and have less satisfaction with social support. The third subgroup of patients have clinical symptoms that are not correlated with HCC or psychosocial stressors. Hence our data suggest mechanistic or biological heterogeneity within the patient population. Psychosocial stressors may contribute to the severity and complexity of a certain subgroup of schizophrenia patients who seem to be vulnerable to stress. This kind of vulnerability indicated by HPA axis dysfunction may be one of the biomarkers of schizophrenia patients since the clinical high risk population also exhibits this tendency (52).

Our study conveys importance messages to help practitioners understanding the role of psychosocial stressors in the etiology and treatment of schizophrenia. Psychiatrist should try to empathize the schizophrenia patients on how much the childhood traumatic events or SLE could have influenced their symptoms, e.g., the severity of delusions (53, 54). Our findings could also be relevant in the treatment of schizophrenia when choosing therapeutic interventions that manipulate cortisol levels (55). Considering the moderating effects of social support for the relationship between SLE and HCC, mental health practitioners should provide more social and psychological support to schizophrenia patients throughout the course of this disease, especially in the stable phase (20, 43, 56). Furthermore, aerobic exercises have been proved to decrease the cortisol levels (57). Taichi is a form of physical exercise and moving meditation originated in China. The role of Taichi and aerobic exercises in the adjunctive treatment of schizophrenia should be investigated further (58).

This study has several strengths. All of the clinical data were collected through face-to-face interviews by trained interviewers with clinical backgrounds. To our knowledge, it is the first study to examine the relationship between psychosocial stressors, social support and HCC in Han Chinese schizophrenia patients.

This study has a number of limitations which should be carefully considered. First, this is a cross sectional study: data were collected retrospectively and recall bias will have affected results. Second, no causal conclusions can be drawn because all the variables were measured only once. Third, we only assessed the significant negative SLE such as the death of significant others and job loss in our study. The influence of everyday living from daily hassles and uplifts which can be measured using the Hassles and Uplifts Scale were not assessed (59). We could not rule out the confounding effects of the above unassessed psychological factors on HPA axis (60). Fourth, we did not examine detailed information on medication for this sample, thus we were not able to adjust for potential effect of medication on HPA axis. Antipsychotic medication may increase (61), decrease (62) or have no influence on cortisol levels in schizophrenia patients (63). Further controlled follow up studies investigating cortisol levels in antipsychotic-naïve patients with schizophrenia treated with certain antipsychotic medication will help to make this clearer.

In summary, our results show HCC are associated with the amount of social support and the severity of delusions in schizophrenia patients. Social support may act as a moderator for the relationship between SLE and HCC which could attenuate the effects of SLE in schizophrenia. We conclude psychosocial stressors and abnormal HCC may contribute to the severity of delusions in a subgroup of stress-vulnerable schizophrenia patients.

## Data Availability Statement

The raw data supporting the conclusions of this article will be made available by the authors, without undue reservation.

## Ethics Statement

The studies involving human participants were reviewed and approved by Ethics Committee of Shanghai Mental Health Center. The patients/participants provided their written informed consent to participate in this study. 

## Author Contributions

Conceived and designed the experiments: FY, HX. Performed the experiments: FY, XS, XC, HW, JQ. Analyzed the data: FY. Wrote the paper: FY. All authors contributed to the article and approved the submitted version. 

## Funding

This work was supported by the Shanghai Jiaotong University Medical-Engineering Cross Fund (YG2015MS48).

## Conflict of Interest

The authors declare that the research was conducted in the absence of any commercial or financial relationships that could be construed as a potential conflict of interest.
